# Durvalumab in advanced biliary tract cancer

**DOI:** 10.1590/1806-9282.2025D721

**Published:** 2026-05-01

**Authors:** Maurício Anhesini, Antonio Silvinato, Idevaldo Floriano, Wanderley Marques Bernardo

**Affiliations:** 1 Brazilian Medical Association, Evidence-Based Medicine – São Paulo (SP), Brazil.; 2 Universidade de São Paulo, Faculty of Medicine – São Paulo (SP), Brazil.

## INTRODUCTION

Biliary tract cancer is a heterogeneous group of malignant neoplasms that includes intrahepatic and extrahepatic cholangiocarcinoma, gallbladder cancer, and carcinoma of the ampulla of Vater. In most cases, diagnosis occurs at advanced stages, when curative surgery is not feasible and the prognosis is unfavorable^1^. Even among patients undergoing potentially curative surgical resection, recurrence and mortality rates remain high: Kaplan-Meier estimates indicate that 57% of cases of extrahepatic cholangiocarcinoma and 61–98% of cases of intrahepatic cholangiocarcinoma result in recurrence or death within 5 years after surgery^2^,^3^. These data reinforce the need for more effective systemic therapies.

Durvalumab is an immunotherapeutic monoclonal antibody approved for the treatment of various solid tumors, including lung, liver, and biliary tract cancers. Its action is based on inhibiting the interaction between the PD-L1 (programmed death-ligand 1) protein, expressed on the surface of some tumor cells, and its PD-1 receptor on T cells. Under physiological conditions, this interaction suppresses the antitumor immune response; by blocking PD-L1, durvalumab restores immunological activity, allowing the immune system to recognize and eliminate neoplastic cells^4,5^.

In this review, we evaluate the efficacy and safety of the combination of durvalumab with gemcitabine and cisplatin (GemCis) as a first-line treatment for patients with advanced biliary tract cancer, including cholangiocarcinoma and gallbladder cancer.

### Clinical question

How effective and safe is durvalumab in the treatment of advanced biliary tract cancer?

### Eligibility criteria

- Patient: Advanced biliary tract cancer (unresectable, locally advanced, or metastatic biliary tract adenocarcinoma, including intrahepatic or extrahepatic cholangiocarcinoma and gallbladder carcinoma).
- Intervention: Durvalumab, either in combination or not.
- Comparison: Placebo.
- Outcomes: Overall survival, response, progression-free survival, adverse events.
- Study design: Randomized controlled clinical trial.
- Period consulted: No limits.
- Full text or abstract with data.

### Information sources

MEDLINE, EMBASE, Google Scholar, and manual search.

### Search strategy

- MEDLINE AND EMBASE

#1 (“Biliary Tract Neoplasms” OR “Biliary Tract Neoplasm” OR “Biliary Tract Cancer” OR “Biliary Tract Cancers” OR “Bile Duct Neoplasms” OR “Gallbladder Neoplasms”)

#2 Durvalumab

#1 AND #2=126

- ClinicalTrials.gov AND Google Scholar

“Biliary Tract Cancer” AND Durvalumab

### Data extraction

- Patient characteristics and interventions.
- Follow-up time.
- Outcomes: Overall survival, response, progression-free survival, adverse events.

### Critical appraisal

- Risk of bias (RoB 2)^6^: Randomization process, intended interventions, missing data, measurement of outcomes, selection of reported results.
- If it were possible to aggregate common results from two or more selected studies, the quality of evidence would be analyzed using GRADE^7^. Otherwise, the quality of evidence would be extrapolated from the risk of bias. Regardless of the source, the quality of evidence would be classified as high, moderate, low, or very low.

### Expression and synthesis of results

- For results expressed by continuous variables (median, etc.), the results (meta-analyzed or not) will be in mean differences.
- For results expressed by categorical variables (percentage, absolute events, etc.), the results (meta-analyzed or not) will be in risk differences.
- The confidence level considered will be 95%
- Random or fixed-effects models were applied based on heterogeneity (≥50% or <50%, respectively). Publication bias was assessed using Egger’s test^8^ whenever possible.

## RESULTS

**Figure 1:**
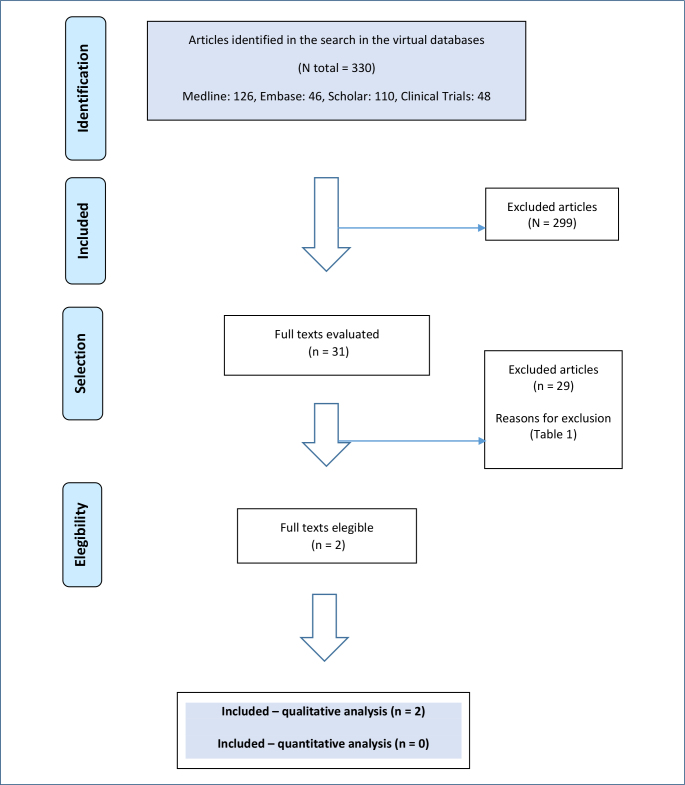
Evidence retrieval and selection diagram: durvalumab and biliary cancer.

The search retrieved a total of 330 publications, distributed across 126 (MEDLINE), 46 (EMBASE), 48 (ClinicalTrials.gov), and 110 (Google Scholar) (Figure 1). After applying the eligibility criteria, two studies were selected, which included the same population but at different follow-up times (1 and 3 years) (Table 1). Excluded studies and the reason for exclusion are in Table 1.

**Table 1:** Summary of study selection and reasons for exclusion.

csv-2AND1-set					
PMID	Title	First author	Year	Included/excluded	Reasons
40611811	Clinical outcomes of transarterial chemoembolization combined with durvalumab for advanced and metastatic biliary tract cancer	Hu Y	2025	Excluded	Case series
40387725	Factors associated with reaching maintenance therapy in patients with advanced biliary tract cancer treated with durvalumab: real-world results from a multicenter and multinational study	Rimini M	2025	Excluded	Prognostic study
40700609	Exploring the Impact of first-line durvalumab plus chemotherapy on advanced biliary tract cancer: a systematic review and meta-analysis	Reimann P	2025	Excluded	Systematic review
40622010	Immune-mediated adverse events in the randomized phase 3 TOPAZ-1 study of durvalumab plus gemcitabine and cisplatin in advanced biliary tract cancer	Antonuzzo L	2025	Excluded	Prognostic study
40533571	Treatment with gemcitabine/cisplatin and durvalumab for advanced biliary tract cancer—Real-world data from a multicenter German patient population	Gerhardt F	2025	Excluded	Real world
40381735	Durvalumab plus chemotherapy in advanced biliary tract cancer: 3-year overall survival update from the phase III TOPAZ-1 study	Oh DY	2025	Included	RCT - 3 years
40765809	Durvalumab-combined chemotherapy for biliary tract cancer in a Japanese expert center: initial 50 cases in daily practice	Inokawa Y	2025	Excluded	Case series
40209292	Molecular targeted maintenance therapy versus standard of care in advanced biliary cancer: an international, randomised, controlled, open-label, phase III umbrella trial (SAFIR-ABC10-precision medicine)	Malka D	2025	Excluded	Precision medicine
40230857	Efficacy and safety of immune checkpoint inhibitors in advanced biliary tract cancer: a real-world study	Zheng Y	2025	Excluded	Real world
39427227	Efficacy of cisplatin-gemcitabine-durvalumab in patients with advanced biliary tract cancer experiencing early vs late disease relapse after surgery: a large real-life worldwide population	Lo Prinzi F	2025	Excluded	Real world
39858096	Initial use experience of durvalumab plus gemcitabine and cisplatin for advanced biliary tract cancer in a Japanese Territory Center	Shionoya K	2025	Excluded	Case series
39647475	Survival outcomes of durvalumab in combination with cisplatin and gemcitabine in advanced biliary tract cancer: real-world results from a single Italian institution	Rimini M	2025	Excluded	Real world
39637344	Gemcitabine, cisplatin, and durvalumab experience in advanced biliary tract cancers: a real-world, multicentric data from India	Muddu VK	2024	Excluded	Real world
39172318	Comparative cost-effectiveness of gemcitabine and cisplatin in combination with S-1, durvalumab, or pembrolizumab as first-line triple treatment for advanced biliary tract cancer	Kashiwa M	2024	Excluded	Cost effectiveness
39566070	Real-world effectiveness and prognostic factors of durvalumab plus chemotherapy in a multicentric cohort with advanced biliary tract cancer	Huang WK	2024	Excluded	Real world
39301763	Efficacy, safety, and differential outcomes of immune-chemotherapy with gemcitabine, cisplatin and durvalumab in patients with biliary tract cancers: a multicenter real-world cohort	Mitzlaff K	2024	Excluded	Real world
39002348	Durvalumab plus gemcitabine and cisplatin in advanced biliary tract cancer: a large real-life worldwide population	Rimini M	2024	Excluded	Real world
38856639	FDA approval summary: durvalumab and pembrolizumab, immune checkpoint inhibitors for the treatment of biliary tract cancer	Casak SJ	2024	Excluded	FDA approval
38823398	Durvalumab or placebo plus gemcitabine and cisplatin in participants with advanced biliary tract cancer (TOPAZ-1): Updated overall survival from a randomised phase 3 study	Oh DY	2024	Excluded	RCT - 2 years
38697156	Durvalumab plus gemcitabine and cisplatin in advanced biliary tract cancer (TOPAZ-1): patient-reported outcomes from a randomised, double-blind, placebo-controlled, phase 3 trial	Burris HA 3rd	2024	Excluded	Post hoc analysis
38691295	Durvalumab plus gemcitabine and cisplatin versus gemcitabine and cisplatin in biliary tract cancer: a real-world retrospective, multicenter study	Rimini M	2024	Excluded	Real world
38416377	Durvalumab plus gemcitabine and cisplatin in patients with advanced biliary tract cancer: an exploratory analysis of real-world data	Olkus A	2024	Excluded	Real world
37452505	Durvalumab plus gemcitabine and cisplatin in advanced biliary tract cancer: an early exploratory analysis of real-world data	Rimini M	2023	Excluded	Real world
36859267	Cost-effectiveness analysis of adding durvalumab to chemotherapy as first-line treatment for advanced biliary tract cancer based on the TOPAZ-1 trial	Zhao Q	2023	Excluded	Cost effectiveness
36844853	Cost-effectiveness analysis of durvalumab plus chemotherapy as first-line treatment for biliary tract cancer	Ye ZM	2023	Excluded	Cost effectiveness
38319896	Durvalumab plus gemcitabine and cisplatin in advanced biliary tract cancer	Oh DY	2022	Included	RCT pivotal
35278356	Gemcitabine and cisplatin plus durvalumab with or without tremelimumab in chemotherapy-naive patients with advanced biliary tract cancer: an open-label, single-centre, phase 2 study	Oh DY	2022	Excluded	Phase II
40608977	The impact of molecular alterations in patients with advanced biliary tract cancer receiving cisplatin, gemcitabine and durvalumab: a large real-life worldwide population	Rimini M	2025	Excluded	Prognostic study
40598212	Prognostic impact of neutrophil-to-lymphocyte ratio (NLR) in patients with unresectable biliary tract cancer treated with gemcitabine, cisplatin, and durvalumab	Mii S	2025	Excluded	Prognostic study
40525496	A prognostic index for advanced biliary tract cancer treated with cisplatin, gemcitabine and durvalumab: the MAGIC-D index	Persano M	2025	Excluded	Prognostic study
39358611	Exploring the impact of durvalumab on biliary tract cancer: insights from real-world clinical data	Reimann P	2024	Excluded	Real world

### Description of included studies

#### Oh etal. (TOPAZ-1 study)^9^

This study included adults (18 years or older) with histologically confirmed, unresectable, locally advanced, or metastatic biliary tract adenocarcinoma, including intrahepatic or extrahepatic cholangiocarcinoma and gallbladder carcinoma, were eligible for inclusion. Eligible patients included those with previously untreated, unresectable or metastatic disease at initial diagnosis, as well as those who developed recurrent disease more than 6 months after surgery with curative intent and more than 6 months after completion of adjuvant therapy. Other inclusion criteria included an Eastern Cooperative Oncology Group (ECOG) performance status of 0 or 1 (on a 6-point scale, where 0 is fully active and 5 is dead), one or more measurable lesions according to Response Evaluation Criteria in Solid Tumors version 1.1 (RECIST v1.1), and no prior exposure to immune-mediated therapy.

TOPAZ-1 was a global, randomized, double-blind, placebo-controlled, phase 3 study. Patients were randomly assigned in a 1:1 ratio to receive durvalumab in combination with gemcitabine and cisplatin (GemCis) or placebo in combination with GemCis. Randomization was stratified by disease status (initially unresectable vs. recurrent) and primary tumor location (intrahepatic cholangiocarcinoma vs. extrahepatic cholangiocarcinoma vs. gallbladder cancer).

Durvalumab or placebo combined with GemCis was administered intravenously in a 21-day cycle for up to eight cycles. Durvalumab (1,500 mg) or placebo was administered on day 1 of each cycle, in combination with gemcitabine (1,000 mg/m^2^) and cisplatin (25 mg/m^2^), which were administered on days 1 and 8 of each cycle. After completion of gemcitabine and cisplatin, 1,500 mg of durvalumab or placebo as monotherapy was administered once every 4 weeks until clinical or radiographic disease progression (according to RECIST v1.1) or until unacceptable toxicity, withdrawal of consent, or if any other discontinuation criteria were met. Patients who were clinically stable at initial disease progression could continue to receive study treatment at the discretion of the investigator and the patient.

Tumor assessments were performed according to RECIST v1.1 using computed tomography or magnetic resonance imaging of the chest, abdomen, and pelvis, and were assessed by the investigator.

Adverse events were reported from the time of informed consent until 90 days after the last dose of study treatment; the causal relationship between reported adverse events and study treatment was assessed by the investigator. Other safety assessments included physical examinations, laboratory findings, ECOG performance status, electrocardiograms, and vital signs.

Health-related quality of life was assessed using the European Organisation for Research and Treatment of Cancer 30-item core quality-of-life questionnaire (EORTC QLQ-C30) with scores ranging from 0 to 100 for each of the symptom scales, functional scales, and global health status. Higher scores on the functional scales and global health status indicate better function and health status, respectively, while higher scores on the symptom scales represent greater symptom severity. Health-related quality of life was also assessed using the 21-item EORTC Quality of Life Questionnaire for Cholangiocarcinoma and Gallbladder Cancer (EORTC QLQ-BIL21), which includes five multi-item domain scales and three single-item scales. For all items and scales, results were scored from 0 to 100, with higher scores indicating greater symptom severity. A clinically meaningful change was defined as an absolute change of 10 or more for these scales (EORTC QLQ-C30 and EORTC QLQ-BIL21).

Patient-reported treatment side effects were assessed using the Patient-Reported Outcomes version of the Common Terminology Criteria for Adverse Events, which consists of nominal categories (e.g., “none” to “very severe” or “not at all” to “very much”).

The primary objective was to assess overall survival (OS), defined as the time between randomization and death from any cause, in the durvalumab group versus the placebo group.

Secondary outcomes included progression-free survival, objective response rate, duration of response, disease control rate, and efficacy by PD-L1 expression, according to RECIST v1.1 using investigator assessments, in the durvalumab versus placebo groups. Progression-free survival was defined as the time from the date of randomization to the date of radiographic disease progression defined by RECIST v1.1 or death.

Safety and side effects were assessed for the durvalumab and placebo groups, with adverse events classified according to the National Cancer Institute Common Terminology Criteria for Adverse Events, version 5.0.

From April 2019 to December 2020, 914 patients were enrolled at 105 sites in 17 countries. In total, 685 patients were randomly assigned to treatment: 341 to the durvalumab group and 344 to the placebo group. Of these patients, 338 and 342 received treatment, respectively.

Patient demographics and disease characteristics were generally balanced between the treatment groups.

## RESULTS

At the data cutoff (August 11, 2021), the median duration of follow-up was 16.8 months (95%CI 14.8–17.7) in the durvalumab group and 15.9 months (95%CI 14.9–16.9) in the placebo group. Furthermore, 275 patients (81.4%) in the durvalumab group discontinued durvalumab and 322 patients (94.2%) in the placebo group discontinued placebo. From the full analysis set, the number of patients who received one or more regimens of subsequent antineoplastic therapy after discontinuation was 145 (42.5%) in the durvalumab group and 170 (49.4%) in the placebo group.

At the time of data cutoff, 198 patients (58.1%) in the durvalumab group and 226 patients (65.7%) in the placebo group had died. OS was significantly longer with durvalumab compared to placebo (hazard ratio, 0.80; 95%CI 0.66–0.97; p<0.021). Median overall survival was 12.8 months (95%CI 11.1–14.0) in the durvalumab group and 11.5 months (95%CI 10.1–12.5) in the placebo group.

The estimated overall survival rates for durvalumab and placebo were 54.1% (95%CI 48.4–59.4) and 48.0% (95%CI 42.4–53.4) at 12 months, 35.1% (95%CI 29.1–41.2) and 25.6% (95%CI 19.9–31.7) at 18 months, and 24.9% (95%CI 17.9–32.5) and 10.4% (95%CI 4.7–18.8) at 24 months, respectively.

The Kaplan-Meier overall survival curve separated at approximately 6 months of treatment, after which there was a clear and sustained separation of the survival curves in favor of the durvalumab group. The hazard ratio (HR) for overall survival was 0.91 (95%CI 0.66–1.26) up to 6 months and 0.74 (95%CI 0.58–0.94) after 6 months. Furthermore, a kernel-type (smoothed) estimate of the hazard function and the associated log-log (event times) versus log(time) plot confirmed a deviation from the proportional hazards assumption.

Median progression-free survival was 7.2 months (95%CI 6.7–7.4) with durvalumab and 5.7 months (95%CI 5.6–6.7) with placebo. The HR for progression-free survival was 0.75 (95%CI 0.63–0.89; p<0.001). The investigator-assessed confirmed objective response rate (sum of complete and partial response rates in patients with measurable disease) was 26.7% (n=341) in the durvalumab group and 18.7% (n=343) in the placebo group (odds ratio, 1.60; 95%CI 1.11–2.31).

The number of patients who achieved a confirmed complete response was 7 (2.1%) with durvalumab and 2 (0.6%) with placebo, and the number of patients who achieved a confirmed partial response was 84 (24.6%) with durvalumab and 62 (18.1%) with placebo. The percentage of patients with an ongoing response at 9 months or more was 32.6% with durvalumab and 25.3% with placebo. The percentage of patients with an ongoing response at 12 months or more was 26.1% with durvalumab and 15.0% with placebo.

The overall survival and progression-free survival benefits observed with durvalumab in combination with gemcitabine and cisplatin were generally consistent across the clinically relevant subgroups analyzed. In patients with a tumor area positivity (TAP) score for PD-L1 of 1% or more, the HR for overall survival with durvalumab versus placebo was 0.79 (95%CI 0.61–1.00). In patients with a TAP score less than 1%, the HR for overall survival with durvalumab versus placebo was 0.86 (95%CI 0.60–1.23).

The safety analysis set included 680 patients who received one or more doses of durvalumab (n=338) or placebo (n=342). The median (range) duration of study treatment was 7.3 months (0.1–24.5) for durvalumab and 5.8 months (0.2–21.5) for placebo. In the durvalumab group, the median (interquartile range) relative dose intensity of durvalumab, gemcitabine, and cisplatin was 100 (93.8–100), 93.8 (82.5–100), and 93.8 (83.3–100), respectively. In the placebo group, the median (interquartile range) relative dose intensity of placebo, gemcitabine, and cisplatin was 100 (95.0–100), 93.8 (82.2–100), and 93.8 (81.3–100), respectively.

Adverse events of any grade occurred in 336 patients (99.4%) in the durvalumab group and in 338 patients (98.8%) in the placebo group. Grade 3 or 4 adverse events occurred in 256 patients (75.7%) in the durvalumab group and in 266 patients (77.8%) in the placebo group. The rate of discontinuation of any component of treatment due to adverse events was 13.0% in the durvalumab group and 15.2% in the placebo group. The number of deaths due to adverse events was 12 (3.6%) in the durvalumab group and 14 (4.1%) in the placebo group. The most common adverse events were anemia (48.2%), nausea (40.2%), constipation (32.0%), and neutropenia (31.7%) in the durvalumab group, and anemia (44.7%), nausea (34.2%), and decreased neutrophil count (31.0%) in the placebo group. Treatment-related grade 3 or 4 adverse events, occurring in 2% or more of patients in the durvalumab and placebo groups. The rate of immune-mediated adverse events was 12.7% with durvalumab and 4.7% with placebo. Grade 3 or 4 immune-mediated adverse events occurred in 2.4% of patients in the durvalumab group and 1.5% in the placebo group.

### Oh etal. (TOPAZ-1 study)^10^

The study methodology and primary efficacy and safety results were previously reported^9^. This update, with analyses of overall survival and safety, long-term survival, and subsequent use of anticancer therapy, occurred with a median follow-up of 41.3 months.

### Updated 3-year exploratory analyses

This study reports overall survival (defined as the time between randomization and death from any cause; or censoring at the last recorded date the participant was known to be alive), characterization of extended long-term survivors (eLTS), subsequent anticancer therapy, and serious adverse events after more than 3 years of follow-up. All analyses presented were exploratory, with no formal statistical testing.

At the data cutoff date (DCO), OS was assessed in the durvalumab arm versus the placebo arm, in the full analysis set (FAS; all randomized participants), according to disease control status {DCS; participants with disease control [complete or partial response (CR/PR; confirmed responses only) or stable disease (SD)] versus progressive disease (PD)} and according to best objective response (BOR; CR/PR vs. SD vs. PD). DCS and BOR were determined by the investigator according to RECIST version 1.1 criteria, based on assessments from the primary analysis, when these analyses were considered final.

## RESULTS

In total, 685 participants were randomized to the durvalumab (n=341) or placebo (n=344) arms. Baseline demographic and clinical characteristics were generally balanced between the treatment arms, as previously reported.

At the DCO, the median follow-up time (95%CI) was 41.3 (39.3–44.1) months in all participants (durvalumab: 42.9 [39.8–44.3] months; placebo: 41.8 [36.7–46.2] months). The data maturity for overall survival was 89.3% overall (durvalumab: 85.9%; placebo: 92.7%), and 13 of 685 (1.9%) participants were still receiving study treatment (all in the durvalumab arm).

After a median follow-up of 41.3 months (95%CI 39.3–44.1) in the FAS (intention-to-treat population), the median (95%CI) OS for durvalumab+GemCis versus placebo+GemCis was 12.9 (11.6–14.1) versus 11.3 (10.1–12.5) months (hazard ratio, 0.74 [95%CI 0.63–0.87]); the 36-month OS rate was 14.6% versus 6.9%, respectively.

Among participants who achieved disease control (566/685; 82.6%), the 36-month OS rate was higher in the durvalumab+GemCis group (17.0%) compared to the placebo+GemCis group (7.6%). Overall, 12.8% were classified as eLTS, with a higher proportion in the durvalumab+GemCis group (17.0%) than in the placebo+GemCis group (8.7%); eLTS included all clinically relevant subgroups. Treatment with durvalumab+GemCis improved OS regardless of subsequent antineoplastic therapy use.

Among eLTS, serious adverse events were comparable between the groups and less frequent than in the safety analysis set (SAS—all participants who received ≥1 dose of study treatment, according to the treatment actually received, regardless of the one to which they were randomized).

### Evidence synthesis

In total, 685 patients with advanced biliary tract adenocarcinoma were randomized to receive durvalumab+gemcitabine/cisplatin (GemCis; n=341) or placebo+GemCis (n=344).

At the primary data cutoff, overall survival (OS) was significantly higher in the durvalumab group compared to placebo (HR 0.80; 95%CI 0.66–0.97; p=0.021). The estimated 24-month OS rate was 24.9% (95%CI 17.9–32.5) versus 10.4% (95%CI 4.7–18.8), respectively. Median progression-free survival (PFS) was 7.2 months (95%CI 6.7–7.4) with durvalumab and 5.7 months (95%CI 5.6–6.7) with placebo (HR 0.75; 95%CI 0.63–0.89; p<0.001). The objective response rate was 26.7% versus 18.7%, and the incidences of grade 3 or 4 adverse events were similar (75.7% vs. 77.8%).

After an extended median follow-up of 41.3 months (95%CI 39.3–44.1), the results confirmed the sustained benefit of durvalumab+GemCis, with a median OS of 12.9 months (95%CI 11.6–14.1) versus 11.3 months (95%CI 10.1–12.5) in the placebo+GemCis group (HR 0.74; 95%CI 0.63–0.87). The 36-month OS rates were 14.6 and 6.9%, respectively. Among participants with disease control (82.6%), the 36-month OS rate was 17.0% with durvalumab+GemCis and 7.6% with placebo+GemCis. Overall, 12.8% of patients were classified as eLTS, with a higher proportion in the durvalumab arm (17.0%) than in the placebo arm (8.7%). The survival benefit was observed regardless of subsequent antineoplastic therapy use. Among eLTS, serious adverse events were comparable between the groups and less frequent than in the safety analysis set.

Collectively, the findings from the 3-year follow-up confirm the durability of the clinical benefit initially observed with durvalumab in combination with GemCis, without a relevant increase in toxicity.

### Risk of bias and quality of evidence

**Figure 2:**
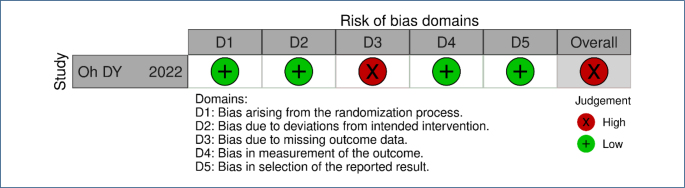
Assessment of risk of bias.

The risk of bias was considered high due to the absence of complete outcome data (Figure 2) and the exploratory nature of the follow-up analyses, which limits the robustness of the conclusions. The overall quality of the extrapolated evidence was classified as low, as the long-term survival analyses were not pre-specified and did not include formal statistical testing.

## DISCUSSION

In most cases of unresectable, locally advanced, or metastatic biliary tract adenocarcinoma, diagnosis occurs at late stages, when curative surgery is not feasible and the prognosis remains unfavorable^1^. Even among patients undergoing potentially curative resection, recurrence and mortality rates remain high^1^.

This systematic review addressed a variety of clinical outcomes, including overall survival, progression-free survival, objective response rate, and incidence of adverse events, offering a comprehensive assessment of the efficacy and safety of durvalumab in combination with gemcitabine and cisplatin (GemCis) in the treatment of advanced biliary tract cancer.

At the primary data cutoff, treatment with durvalumab resulted in a significant improvement in OS compared to placebo (HR 0.80; 95%CI 0.66–0.97; p=0.021), with 24-month OS rates of 24.9% versus 10.4%, respectively. Median PFS was 7.2 months (95%CI 6.7–7.4) with durvalumab and 5.7 months (95%CI 5.6–6.7) with placebo (HR 0.75; 95%CI 0.63–0.89; p<0.001). The objective response rate was higher in the durvalumab group (26.7 vs. 18.7%), while the incidences of grade 3 or 4 adverse events were similar between the groups (75.7 vs. 77.8%)^9^.

After a median follow-up of 41.3 months, the overall survival benefit was maintained (HR 0.74; 95%CI 0.63–0.87), with 36-month OS rates of 14.6% in the durvalumab group and 6.9% in the placebo group. Among patients who achieved disease control, the 36-month OS rates were 17.0 and 7.6%, respectively. Patients classified as eLTS accounted for 12.8% of the total population, being more frequent in the durvalumab group (17.0%) than in the placebo group (8.7%). Serious adverse events among eLTS were comparable between the groups and occurred less frequently than in the full SAS^10^.

In summary, our analysis indicates that the addition of durvalumab to gemcitabine and cisplatin provides a relevant and sustained clinical benefit in the overall survival of patients with advanced biliary tract cancer, without a substantial increase in toxicity. The survival gain was consistent after 3 years of follow-up, reflecting a higher proportion of long-term survivors in the durvalumab arm.

However, it is necessary to acknowledge the limitations inherent in the available evidence. The risk of bias was considered high due to the absence of complete outcome data and the exploratory nature of the follow-up analyses, which reduces the robustness of the conclusions. Furthermore, the overall quality of the extrapolated evidence was classified as low, as the long-term survival analyses had not been pre-specified in the study protocol and did not include formal statistical testing.

## CONCLUSION

In the treatment of patients with advanced biliary tract cancer, the use of durvalumab in combination with GemCis, compared to GemCis alone, reduced mortality (24 months) by 14.5% (number needed to treat of 7), without a difference in serious adverse events. This benefit was maintained over 36 months of follow-up.

The quality of evidence was considered low, due to the absence of complete outcome data and the exploratory nature of the follow-up analyses.

## Data Availability

The datasets generated and/or analyzed during the current study are available from the corresponding author upon reasonable request.
